# Enhancing Patient Identification Accuracy in Shared Child Health Records: a Hybrid Approach for the Lao Language Context

**DOI:** 10.1007/s10916-025-02260-6

**Published:** 2025-09-26

**Authors:** Thepphouthone Sorsavanh, Chang Liu, Goshiro Yamamoto, Yukiko Mori, Shinji Kobayashi, Tomohiro Kuroda

**Affiliations:** 1 Medical Informatics Division, Graduate School of Informatics, Kyoto University, Yoshida-Honmachi, Sakyo Ward, Kyoto, 606-8501 Japan; 2 Medical Information Technology and Administration Planning, Kyoto University Hospital, 54 Shogoin Kawaracho, Sakyo Ward, Kyoto, 606-8507 Japan; 3 Preemptive Medicine and Lifestyle Related Disease Research Center, Kyoto University Hospital, 54 Shogoin Kawaracho, Sakyo Ward, Kyoto, 606-8507 Japan; 4 School of Medicine, Gifu University, 1-1 Yanagido, Gifu, 501-1194 Japan

**Keywords:** Patient identification systems, Algorithms, Health information exchange, Lao people’s democratic republic

## Abstract

The Shared Child Health Record (SCHR) project in Lao People’s Democratic Republic (PDR) aims to enhance pediatric health care services and health outcomes by enabling data exchange between health care systems. However, persistent challenges of duplication due to patient identification are hindered by non-Latin script complexities, including phonetic variations, a tonal alphabet, and temporary naming practices (e.g., placeholder names such as “Eanoi”). Existing patient-matching algorithms designed for Latin scripts underperform in this context. We assessed deterministic, probabilistic, and hybrid matching approaches using a Lao SCHR dataset of 20,433 records. A manual gold standard review (3,191 matches) validated their performance. Probabilistic matching employed the Fellegi–Sunter model with Jaro‒Winkler similarity, whereas the hybrid method combined deterministic rules (exact name/DOB matches) and probabilistic adjustments for unresolved cases. The hybrid and probabilistic methods consistently outperformed deterministic matching, achieving a 90% recall rate on the SCHR dataset. Despite its lower performance in Lao health records, the hybrid method resolved approximately 2,872 duplicates in SCHR. Challenges included twin records (shared identifiers) and temporary-to-permanent name transitions. This study is the first to adapt patient-matching methodologies for Lao’s linguistic and infrastructural context. While hybrid methods show promise, performance gaps persist compared with those of Latin-based systems. These findings have significant implications with respect to improving the accuracy and efficiency of HIE systems in Lao PDR and other resource-limited settings.

Clinical trial number: Not applicable.

## Introduction

We studied patient matching in Lao script for shared child health records, where cross-facility data exchange makes linkage essential. While methods exist for other languages, there is no Lao-specific approach; we adapt and evaluate a Lao-tailored workflow. Health information exchange (HIE) offers considerable benefits to health care systems, especially by facilitating data sharing between health care entities and improving both patient and provider outcomes [1]. In Lao People’s Democratic Republic (PDR), the Shared Child Health Record (SCHR) connects the electronic immunization record system (e.g., District Health Information Software Version 2) and the electronic hospital record system, enabling electronic exchange of patient data to foster trust among health care providers and enhance immunization coverage [2]. However, the pilot project revealed critical data quality issues, including duplication, incomplete records, and patient mismatches, primarily attributed to the absence of standardized patient demographic data and frequent data entry errors.

An environmental scan reveals that while patient-matching algorithms have advanced for Latin-based languages, non-Latin scripts such as Lao remain underserved, facing unique hurdles due to Lao’s tonal alphabet, phonetic variations, and temporary naming practices (e.g., placeholder names such as “Eanoi”). The Lao script, with 27 consonants, 6 tones, some common Lao names (e.g., ເອື້ອຍ [Eoui] → nickname “baby”) and their Latin transliterations and compound characters (e.g., ສຸກສັນ (Souksan) vs. ສູກສັນ (Souksan)), introduces ambiguities that are absent in Latin systems. Compounded by infrastructural barriers—such as rural–urban disparities, limited internet access, and decentralized health care—these issues result in duplicate records, incomplete data, and safety risks. Reliable patient identification is essential within HIE systems, where patient data movement across organizations amplifies the need for accurate patient matching [3].

Studies by Gupta et al. [4] and McCoy et al. [5] have highlighted the challenges of fragmented and incomplete patient records, particularly in environments lacking a unique patient identifier. Issues such as data duplication and mismatches can result in inefficient care, safety risks, and increased costs. Gupta et al. emphasized the limitations of probabilistic and heuristic matching algorithms in achieving accuracy when data quality and standardization issues are poor. Similarly, McCoy et al. discussed the prevalence of duplicate records within health care systems and the associated risks, emphasizing the need for effective management strategies to address these issues. In Japan, the National Database of Health Insurance Claims anonymizes patient data using two identifiers (ID1 and ID2); however, life events and clerical errors hinder longitudinal tracking. To address this, a virtual patient identifier has been implemented, merging both identifiers to enhance patient traceability over time [6]. Similarly, in Australia, integrating health data from multiple sources has enabled monitoring and assessment of childhood immunization programs. This data integration not only offered a comprehensive overview of program effectiveness but also revealed the challenges of merging large datasets from different jurisdictions, particularly in terms of record structure, quality, and completeness [7].

Various patient matching and linkage algorithms have been developed and implemented globally [3, 8–11]. These include the following: (1) deterministic matching uses rules-based processes for record exacting; (2) probabilistic matching [8] computes match weights for attributes and utilizes methods such as bloom filters and naïve Bayes; (3) machine learning algorithms encompass both supervised and unsupervised techniques, utilizing k-means clustering, semantic matching (which achieves the highest quality when multiple similarity measures are combined into a single measurement system), and similarity measurements; (4) machine learning approaches encompass statistical matching, propensity score matching, regression-based matching, and nearest neighbor methods; (5) hybrid methods integrate the strengths of various record linkage approaches, particularly deterministic and probabilistic methods, to achieve accuracy and precision; (6) privacy-preserving record linkage (PPRL) is a data integration technique that facilitates the linking of disparate datasets while maintaining data confidentiality; and (7) reference matching utilizes large external demographic datasets to enhance the matching process by providing a more comprehensive view of a patient’s identity. Numerous applications are available for record matching, with several community organizations providing relevant tools. The open-source packages on GitHub [12] include Open Client Registry (OpenCR), Open Enterprise Master Patient Index (OpenEMPI), SPLINK (probabilistic record linkage), PySyft (privacy-preserving federated learning), RecordLinkage (Python Library for record linkage), Entity Resolution with Dedupe (Python Library), and the PPRL Toolkit. While these methods excel in Latin contexts, their direct application to Lao’s linguistic and infrastructural landscape is unproven. This study adapts and evaluates these techniques for Lao’s SCHR, addressing gaps in non-Latin HIE systems. By resolving duplicates and improving data quality, this work offers a blueprint for low-resource regions facing similar challenges, from Southeast Asia to sub-Saharan Africa.

## Methods

The system was tested in Borikhamxay Province, Lao PDR (population: 330,744; 50 health facilities), where geographical barriers (mountainous terrain, rural‒urban divides) and infrastructural gaps (limited internet) fragment data collection. Despite standardized protocols nationwide, cultural practices (e.g., temporary names) and decentralized systems cause inconsistencies in patient matching. These systemic challenges—not regional differences—highlight universal hurdles in Laos’ resource-limited context.

**Fig. 1:**
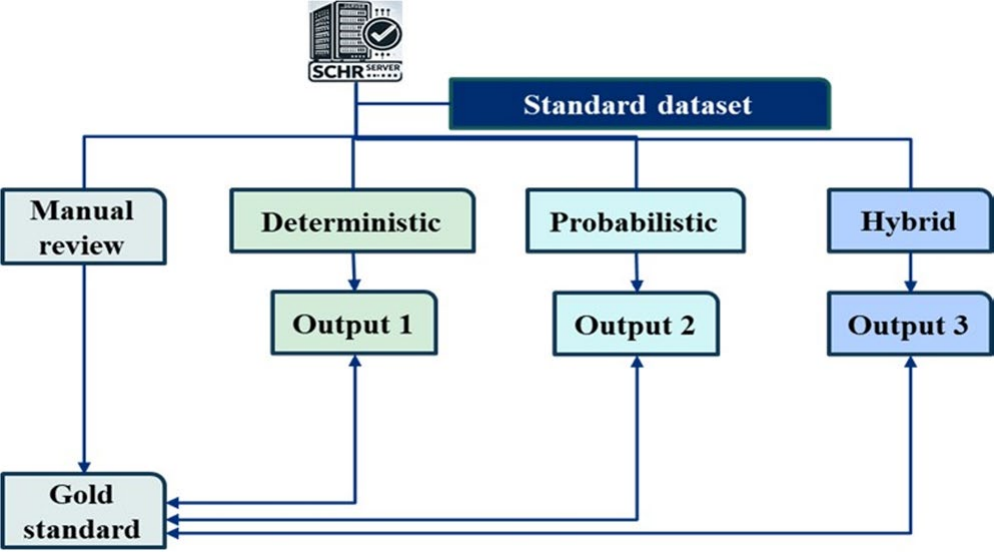
Study procedures: (1) use a standard dataset, (2) perform manual matching as the gold standard, (3) apply matching techniques, and (4) conduct comparative metric analysis

This study employed a multistep approach to compare three patient-matching techniques—deterministic, probabilistic, and hybrid matching—using a standard dataset derived from the SCHR server in Lao PDR. A manual review and validation process were carried out to establish a gold standard, which served as the benchmark for assessing the accuracy and efficiency of the three techniques. This thorough evaluation offered insights into which approach performs most effectively in the context of HIE (Fig. 1).

### Standard Dataset

A standard dataset is essential for patient matching, as demographic attributes are consistently collected across all health care facilities, and consistent time periods provide a foundation for reliable patient matching [13]. Data preparation involved the use of blocking variables, such as names, birthdates, and gender, to standardize fields. This process included converting text to uppercase, eliminating spaces, and handling accents or special characters [14]. The dataset used for matching comprised columns related to names, date of birth (DOB), sex, and village. Standardization of these fields ensures consistency across datasets. The contact numbers complied with Lao PDR phone formats and were no longer than 50 characters. Some special Lao alphabets were also converted to the standard format [15].

### Manual Review as the Gold Standard

This study employed five eight-point frameworks to generate gold standard datasets for assessing the performance of patient-matching algorithms. This approach ensures accurate matching, minimizes computational complexity, and facilitates comprehensive training [4]. Three reviewers evaluated patient matching using Excel and pivot tables. They identified matching clusters on the basis of DOB, sex, and village while also assessing name and parent name matching. A total of 21 match and nonmatch criteria were established (Table 1) (e.g., name + DOB + village = match).

**Table 1:** 10 match and 11 nonmatch criteria, selected on the basis of the properties of the dataset

Criteria	DOB	Sex	Village	Name	Father’s Name	Mother’s name	Matching
1	match	match	match	match	match	match	TRUE
2	match	match	match	match	match	Non match	TRUE
3	match	match	match	match	Non match	match	TRUE
4	match	match	match	match	Non match	Non match	FALSE
5	match	match	match	Non match	match	match	TRUE
6	match	match	match	Non match	Non match	match	FALSE
7	match	match	match	Non match	match	Non match	FALSE
8	match	match	match	Non match	Non match	Non match	FALSE
9	match	match	match	match	match	Not available	TRUE
10	match	match	match	match	Not available	match	TRUE
11	match	match	match	match	Not available	Not available	TRUE
12	match	match	match	Not available	match	Not available	TRUE
13	match	match	match	Not available	Not available	match	TRUE
14	match	match	match	Not available	match	match	TRUE
15	match	match	match	Not available	Not available	Not available	FALSE
16	match	match	match	match	Non match	Not available	FALSE
17	match	match	match	match	Not available	Non match	FALSE
18	match	match	match	Non match	match	Not available	FALSE
19	match	match	match	Not available	match	Non match	FALSE
20	match	match	match	Not available	Non match	match	FALSE
21	match	match	match	Non match	Not available	match	FALSE

Training: Reviewers underwent a training session on Lao naming conventions (e.g., temporary names such as “ແອນ້ອຍ Eanoi” vs. permanent names). Consensus: Discrepancies (e.g., phonetic variations in ສຸກ [Souk] vs. ສູກ [Souk]) were resolved through discussion.

### Selection of the Optimal Matching Technique

We independently implemented deterministic, probabilistic, and hybrid matching processes and compared their outcomes against the gold standard dataset. Deterministic matching used the same criteria as manual review. We prepared the data and subsequently conducted an exact match based on the child’s name and parents’ names; for example, records for BUPPHA (01/01/2020, Village A) were linked only if all fields matched exactly.

For probabilistic matching, we utilized SPLINK, employing the Fellegi–Sunter linkage model, which facilitates efficient and rapid execution [16]. Exact matching was performed to compare fields such as names and dates of birth, whereas the expectation–maximization algorithm was used to estimate match and nonmatch probabilities. The Jaro–Winkler similarity algorithm was employed to account for minor variations in names and addresses. Blocking criteria based on “DOB” and “village” were implemented to limit comparisons and enhance processing efficiency. The recall for blocking was adjusted from 0.65 to 0.95, achieving an optimal recall of 0.7. Pairwise predictions were utilized to group records into clusters that represent a single entity.

The hybrid matching approach combines deterministic and probabilistic methods. We applied probabilistic matching only to records unmatched deterministically, whereas deterministic matching criteria such as DOB, sex, village, name, and parents’ names were used to validate matches identified probabilistically. This approach ensured that the probabilistic algorithm did not reprocess records that had already been matched by the deterministic method. Both methods treated those records as a match. A study on the Colorado Congenital Heart Disease surveillance system demonstrated how deterministic methods require exact matches for identifiers, whereas probabilistic methods allow for variations and calculate match scores on the basis of the likelihood of linking records [9].

### Evaluation

The performance of the three patient-matching methods—deterministic, probabilistic, and hybrid—on the Lao dataset was evaluated in terms of precision, recall, and F1 score.

## Results

### Demographic Data

**Fig. 2:**
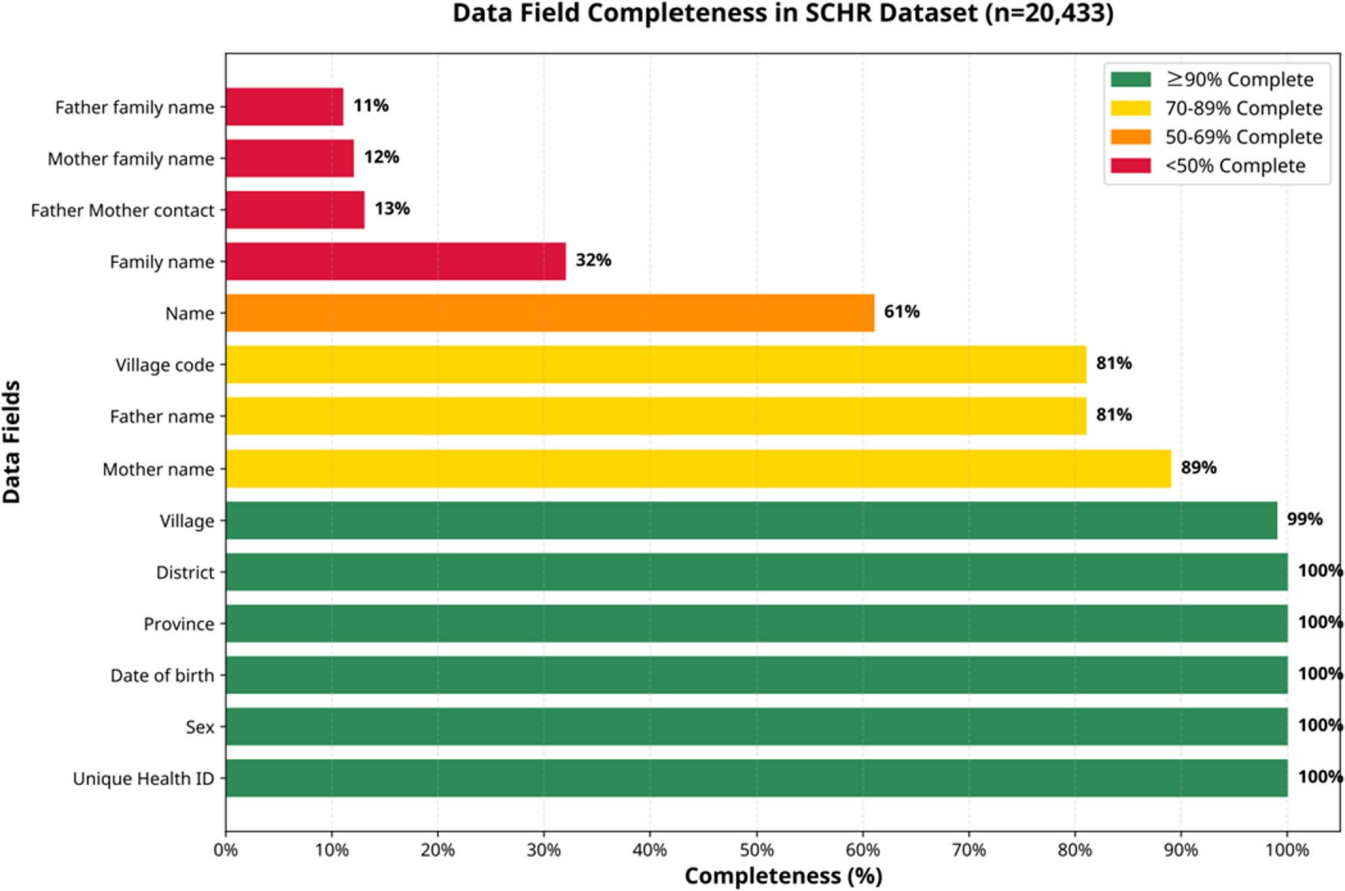
Comprehensiveness of the matching variables used for analysis, including DOB, gender, village, mother’s name, father’s name, and children’s names

The dataset analyzed comprised 20,433 records. We assessed the completeness of the variables within the dataset, with values ranging from 11 to 100%. Incomplete variables that were incomplete were excluded from further analysis, including family name (32%), contact information of the father or mother (13%), and family names of the father and mother (11–12%) (Fig. 2).

**Fig. 3:**
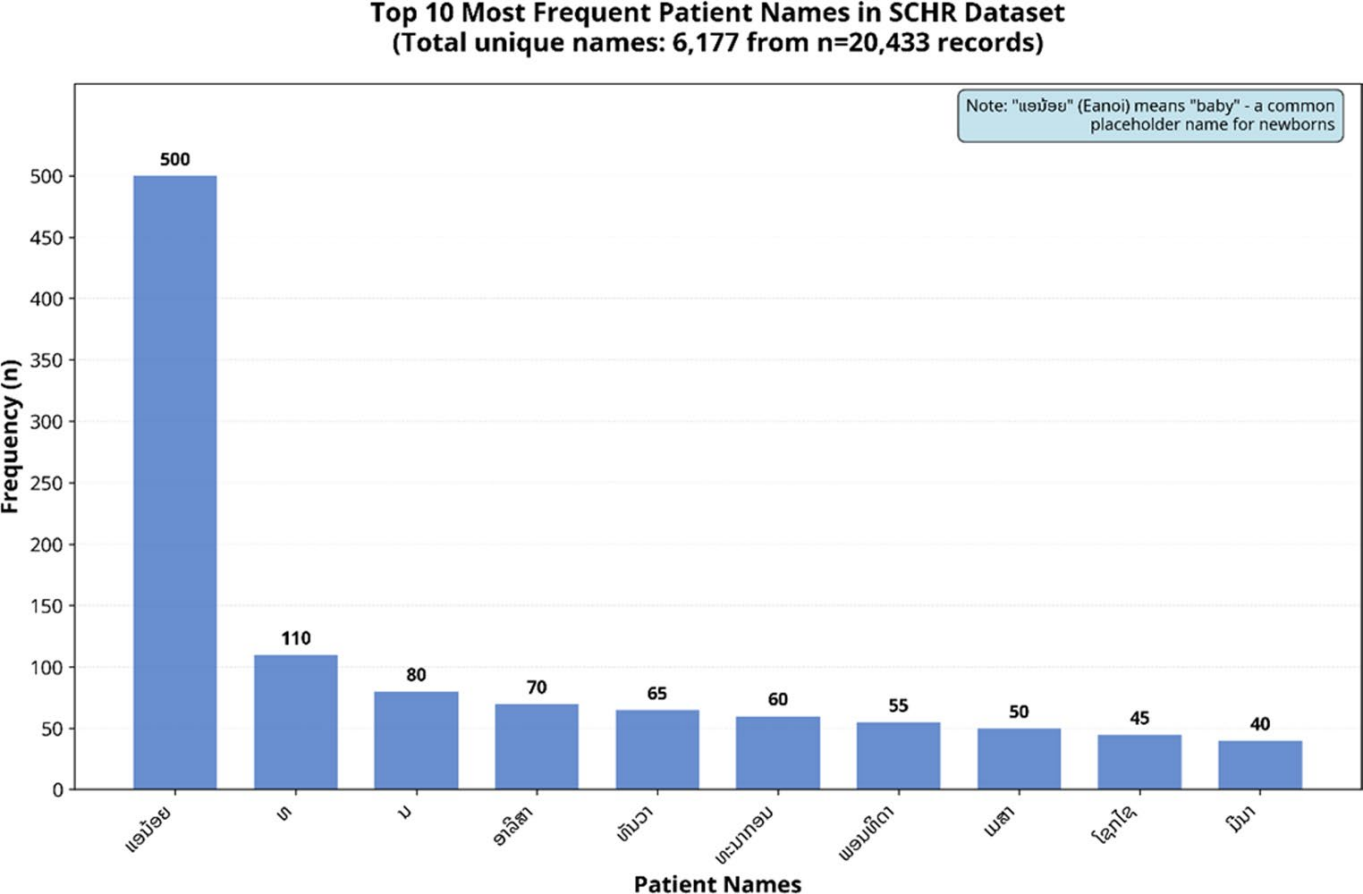
Analysis of the top ten and bottom five values in the “Name” aimed to identify the most and least frequent entries

We examined the distribution of values in the “Name” column, focusing on the frequency of distinct values and missing entries. Frequently occurring names (top 10) were identified, with Eanoi (a nickname meaning “baby”) appearing approximately 500 times, followed by “Mr.” and “Miss” (110 and 80 times, respectively). In contrast, the least frequent names (bottom five) represented permanent names (Fig. 3).

### Manual Review (Gold Standard)

We calculated the ratio of matches to nonmatches identified through a combination of DOB, sex, and village. A total of 5,740 potential matches (28.09%) were flagged for manual review. Three reviewers assessed the matches and nonmatches within these 5,740 records. Reviewer 1 identified 3,409 matches (16.68%), Reviewer 2 identified 3,311 matches (16.2%), and Reviewer 3 identified 3,240 matches (15.86%). Compared with individual reviewers, the final consensus of the gold standard matches resulted in a marginally reduced match rate, with a total of 3,191 matches detected (15.62%) (Table 2).

**Table 2:** Results of a review process, evaluated by three reviewers and considered the gold standard

Reviewer	Total	Match cluster	Nonmatch		Match	
			*n*	%	*n*	%
Reviewer 1	20,433	5,740	17,025	83.32	3,409	16.68
Reviewer 2	20,433	5,740	17,123	83.8	3,311	16.2
Reviewer 3	20,433	5,740	17,194	84.14	3,240	15.86
Agreement 1, 2, and 3 (Gold standard)	**20**,**433**	**5**,**740**	**17**,**242**	**84.38**	**3**,**191**	**15.62**

The breakdown of matching pairs, which is based on the number of records that are matched collectively, yielded several groupings. Most of the matching pairs consisted of two records, representing 86.9% of the total (1,290 pairings out of 1,484). A smaller proportion of matching pairs consisted of three (11.4%), four (1.4%), and five records (0.3%). The largest matching pair comprised 2,580 records, with other groupings consisting of 507, 84, and 20 records (Table 3).

**Table 3:** The distribution of matching pairs among several groupings

	Number of matching pairs				
	2	3	4	5	Total
Number	1,290	169	21	4	1,484
%	86.9%	11.4%	1.4%	0.3%	100
Total matching pairs	**2**,**580**	**507**	**84**	**20**	**3**,**191**

### Performance of Matching Techniques

**Fig. 4:**
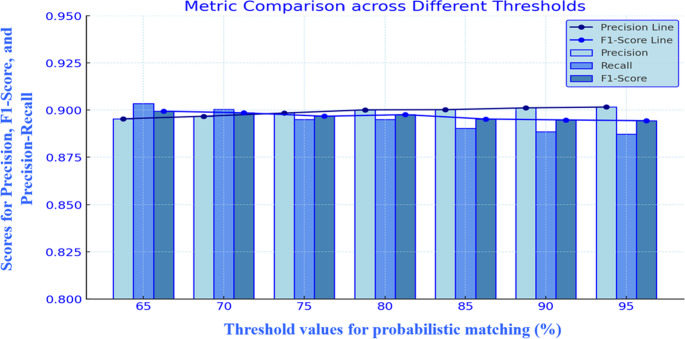
Adjustment of the blocking matching sensitivity for DOB and villages from 65–95% in the probabilistic matching technique and comparison of its performance with that of manual matching

The recall of the blocking matching criterion was adjusted from 65 to 95%, revealing that increasing thresholds improved precision but reduced recall. The model consistently achieved a high F1 score, with values between 65% and 75%. We selected a threshold of 70% for further evaluation (Fig. 4).

**Fig. 5:**
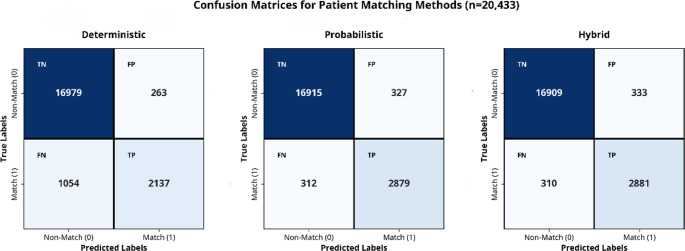
Confusion matrix for three matching techniques applied to the SCHR dataset, including true negatives, true positives, false negatives, and false positives

**Fig. 6:**
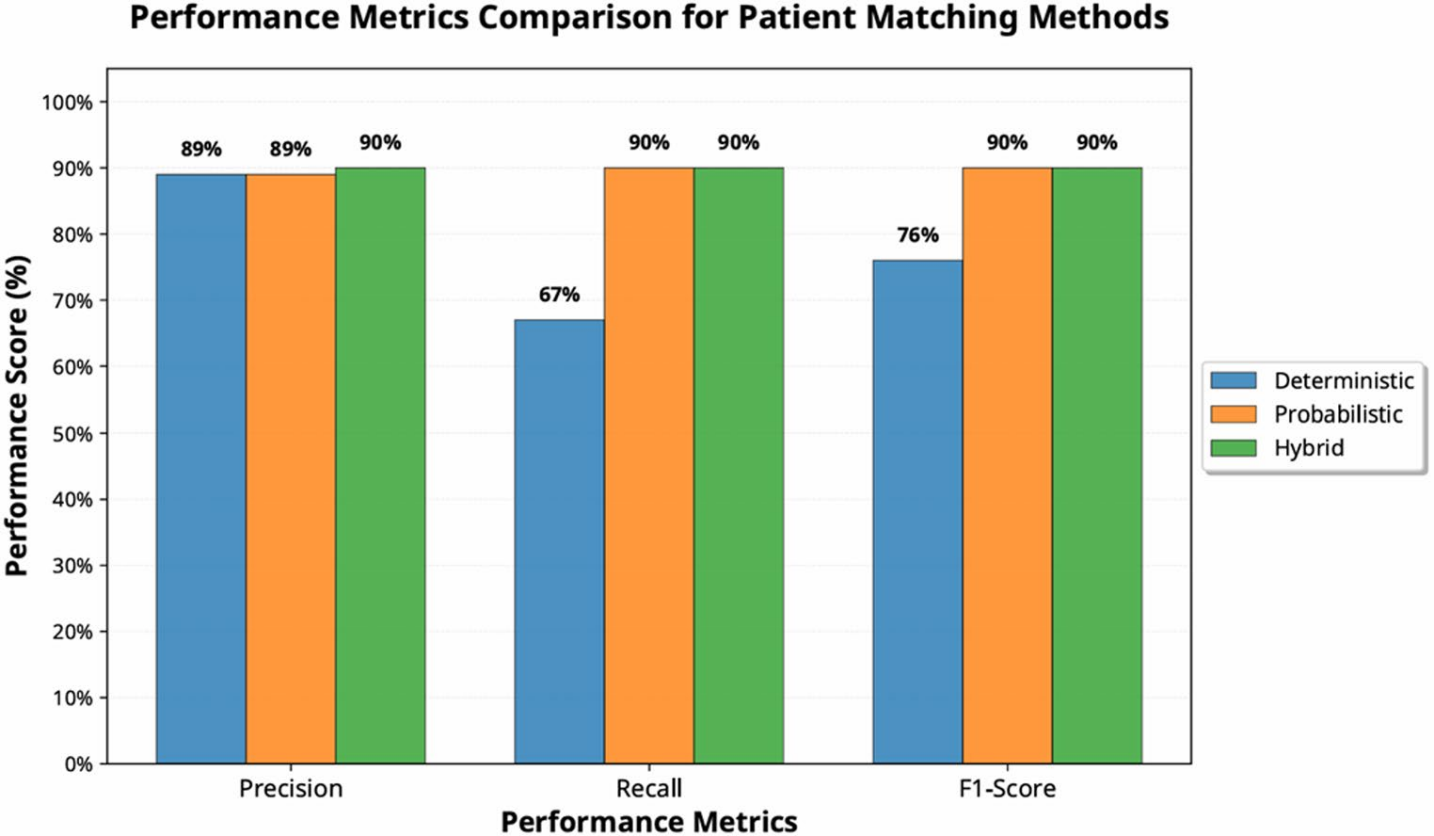
Comparison of precision, recall, and F1 score using the SCHR dataset across three methodologies: deterministic, probabilistic, and hybrid

Figure 5 presents the confusion matrix comparing the performance of three matching methods: deterministic, probabilistic, and hybrid. According to the gold standard, the methods identified 16,979, 16,915, and 16,909 records, respectively, as true negatives; 263, 327, and 333 records as false positives; 1,054, 312, and 310 records as false negatives; and 2,137, 2,879, and 2,881 records as true positives. The precisions of the three methods are notably similar, reaching approximately 89–90%. However, differences are observed in the recall and F1 scores. The probabilistic and hybrid methods outperformed the deterministic method in terms of matching performance, achieving recall rates of 90%, 90%, and 67%, respectively, and F1 scores of 90%, 90%, and 76%, respectively (Fig. 6).

**Fig. 7:**
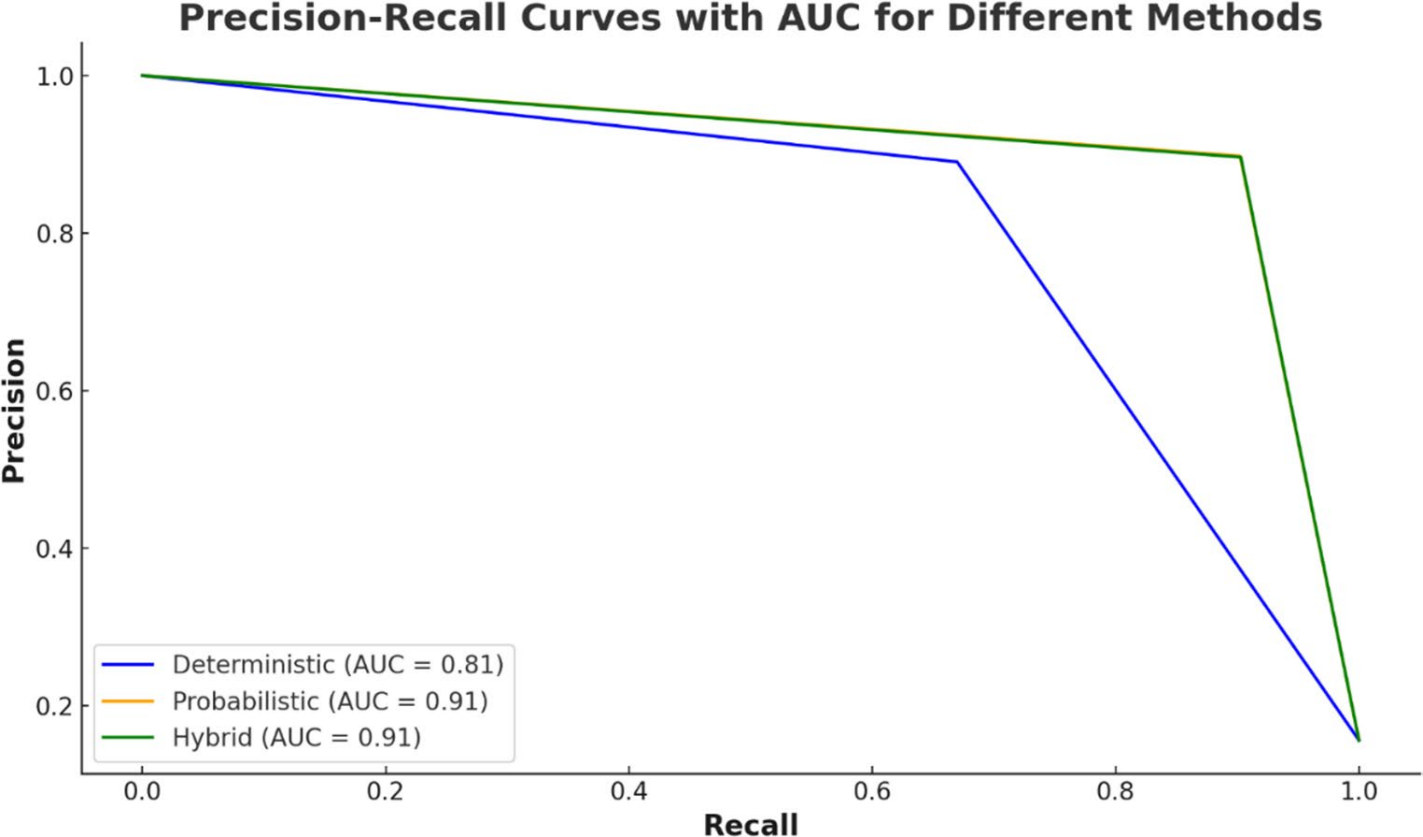
The combined precision‒recall curves plotted using the new data, including the AUC values for the deterministic, probabilistic, and hybrid methods (presented separately)

The precision‒recall curve provides a visual assessment of model performance by illustrating the trade-off between precision and recall across different threshold settings. The deterministic model, with an area under the curve (AUC) of 0.81, initially demonstrated high precision. The precision sharply decreased as the recall increased, indicating a significant trade-off. Compared with the deterministic model, the probabilistic and hybrid models achieved an AUC of 0.91, indicating superior flexibility. These methods provide a better balance between precision and recall (Fig. 7).

## Discussion

This study demonstrates that hybrid patient-matching methods achieved 90% recall in Lao’s SCHR system, outperforming deterministic approaches (67%) by accommodating phonetic variations and temporary naming practices. Despite their effectiveness, these methods demonstrated lower performance than did previously conducted studies using Latin datasets. Examples include systems with high levels of accuracy in matching fingerprint templates [17], PPRL [18], hybrid [9] and reference matching [10]. The unique characteristics of the Lao language—distinctive alphabet, tonal features, and phonetic complexity—contributed to this disparity by reducing the effectiveness of existing algorithms when applied to Lao data. Data quality issues, such as missing family names and parental contact details, further constrained deterministic matching. These gaps stem from Lao PDR’s decentralized health care infrastructure, where rural facilities often lack resources for consistent data entry. While biometric identifiers (e.g., fingerprints [17]) can resolve duplicates, their implementation is impractical in Lao PDR because of cost barriers and the absence of national ID systems. Instead, hybrid methods—leveraging probabilistic flexibility after applying deterministic rules—proved most viable. Notably, the study’s methodology offers broad applicability. For example, tonal languages (e.g., Thai, Cambodian) could adopt similar hybrid frameworks with phonetic encoding. Low-resource settings with decentralized data may replicate the blocking strategy (DOB + village) to improve efficiency.

To increase patient matching accuracy for Lao script—a non-Latin, tonal alphabet—a tailored algorithm that integrates phonetic and structural adaptations could draw inspiration from methodologies developed for other scripts. For example, the Chinese “sound shape code,” which combines phonetic and visual components, minimizes false nonmatches in character-based systems [19]. Similarly, modified phonetic algorithms such as the Spanish Metaphone, optimized for handling spelling variations in Latin scripts, demonstrate how linguistic adjustments improve precision [20]. In resource-constrained settings, tools such as Italy’s SALI software highlight the importance of balancing sensitivity and portability [21], whereas India’s linguistic adaptations of Soundex underscore the value of customizing phonetic rules to reduce errors in large databases [22]. Even for scripts with unique complexities, such as Myanmar’s abugida system, Soundex-inspired encoding has proven effective for name matching [23]. Collectively, these examples emphasize that script-specific adaptations—accounting for tonal, phonetic, and structural nuances—are critical for optimizing patient-matching algorithms. Applying these principles to Lao’s linguistic context could address current gaps in HIE systems, particularly in resolving challenges such as temporary naming conventions and tonal ambiguities.

A critical limitation is that children often do not have a permanent name at birth. Initially, the system records the parent’s name or a placeholder such as “baby,” but the record is updated when the child is assigned a permanent name later. This process can result in discrepancies and incorrect matches, as the system may fail to correctly associate these two entries with each other. A major limitation of the deduplication methods used in this study is their inability to differentiate between the records of twin children. Twins often share key identifiers, such as DOB, location, parent names, and often sex, leading to misclassification as duplicates. While the third algorithm was applied to analyze the Lao dataset, other algorithms not assessed in this study may prove more effective, especially when addressing non-Latin scripts. The manual review processes presented significant challenges, especially in achieving consensus on matching decisions when encountering different spellings—a common characteristic of ethnic group names. This variability added further complexity in identifying true matches.

Future research should investigate interventions aimed at improving patient matching for deduplication in the Lao dataset by incorporating hybrid algorithms, such as Soundex, string matching, and biometric identifier techniques. Furthermore, exploring the development or implementation of a national health ID system could address current challenges in patient identification and further streamline data deduplication processes.

## Conclusion

This study advances patient-matching methodologies for non-Latin scripts by validating a hybrid approach in Lao PDR’s SCHR system. Key contributions include hybrid method efficacy: Combining deterministic rules and probabilistic adjustments resolved 2,872 duplicates, achieving 90% recall despite data incompleteness. Script-Specific Challenges: Highlighting how Lao’s tonal alphabet and temporary names necessitate tailored solutions, diverging from Latin-based systems. Framework for Low-Resource Settings: Demonstrated that blocking strategies (e.g., village + DOB) and phonetic adaptations can increase HIE accuracy in decentralized environments. While the hybrid method shows promise, persistent challenges—such as twin differentiation and manual review biases—call for algorithmic innovations (e.g., Lao Soundex) and infrastructure investments (e.g., unique IDs). These findings extend beyond Lao PDR, offering a blueprint for regions with non-Latin scripts or fragmented health care data. Future research should prioritize phonetic algorithms for tonal languages and integrate temporal–spatial identifiers to address twin-related mismatches.

## Data Availability

The data that support the findings of this study are not openly available for reasons of sensitivity and are available from the corresponding author upon reasonable request. The data are stored in a controlled access data storage facility at the Ministry of Health, Lao PDR. https://moh.gov.la/index.php/download/dataset_analysis_csv/.

## Data Availability

The custom code used to analyze the data and generate the findings of this study is available from the corresponding author upon reasonable request. The code is implemented in Jupyter notebooks and includes documentation for reproducibility.
